# Demographic, Clinical Profile and Outcomes of Neonates Admitted to Neonatal Intensive Care Unit of Dekemhare Hospital, Eritrea

**DOI:** 10.1186/s12887-022-03779-0

**Published:** 2022-12-15

**Authors:** Hailemichael Gebremariam, Berhe Tesfai, Seltene Tewelde, Samsom Abay, Danait Tekeste, Fitsum Kibreab

**Affiliations:** 1 Dekemhare Hospital, Zoba Debub, Ministry of Health, Dekemhare, Eritrea; 2 Massawa Hospital, Northern Red Sea, Ministry of Health, Massawa, Eritrea; 3 Hazhaz Hospital, Zoba Maekel, Ministry of Health, Asmara, Eritrea; 4 Pediatrician, Mendefera Zonal Referral Hospital, Zoba Debub, Ministry of Health, Asmara, Eritrea; 5 Statistician, Ministry of Health, Debub Branch, Mendefera, Eritrea; 6 Epidemiologist, Ministry of Health, Health Research and Resource Center Division, Asmara, Eritrea

**Keywords:** Neonatal, Mortality, Outcomes, Prematurity, Eritrea

## Abstract

**Background:**

Ninety-six percent of the world’s 3 million neonatal deaths occur in developing countries where the majority of births occur outside health facility. The objective of this study was to evaluate the demographic, clinical profile and outcome of neonates admitted to Neonatal Intensive Care Unit of Dekemhare Hospital of Eritrea.

**Methods:**

The study was a retrospective register-based review of all neonates admitted from January 2018 to December 2021 to Dekemhare Hospital. Overall, 509 neonates were enrolled in this study. Data were collected from neonatal register book from January 5 to February 5, 2022 by general practitioners using a predesigned data collection tool. Data entry was done using CSpro 7.3 and analyzed through SPSS version 22. Results were presented in frequencies, percent and odds ratio. Univariable and multivariable analysis was done to measure the association between the variables.

**Results:**

Three quarter (75.6%) of the neonates had normal birth weight and 80.0% were term. Majority (75.4%) of the neonates was delivered vaginally and 92.7% were delivered at health facility. Neonatal infection (33.0%), birth asphyxia (20%) and prematurity (14.3%) were the top three primary causes of neonatal admission to the Neonatal Intensive Care Unit. Furthermore, 31% of neonatal deaths occurred during 24-72 hours of their life and the mortality rate was 16.3%. Multivariable analysis indicated that low birth weight (AOR: 7.28; 95%CI: 2.85-18.55) increased neonatal mortality. Whereas delivery at health facility (AOR: 0.17; 95%CI: 0.06–0.47), hospital stay 4-7 days (AOR: 0.06; 95% CI: 0.02-0.23) and above 8 days (AOR: 0.06; 95%CI: 0.02-0.23) were showing protective effect on neonatal mortality.

**Conclusion:**

Congenital abnormality**,** prematurity and birth asphyxia had higher case fatality rate. And, low birth weight, delivery at health facility and hospital stay were found to be predictors of neonatal mortality. Training of health professionals on neonatal resuscitation, further improvement on the diagnostic setup, treatment tools, infrastructure and raising community awareness to deliver at health facility are crucial to decrease the neonatal mortality in Eritrea.

## Introduction

The neonatal mortality accounts almost two third of the infant mortality rate and 47% of under-five mortality rate worldwide [1, 2]. Worldwide around 2.4 million neonates died in the year of 2019 with neonatal mortality rate of 17 per 1000 live birth [1]. Globally, the neonatal mortality rate fell by 52% from 1990 to 2019 and two regions account for almost 80% of the newborn deaths in 2019; Sub-Saharan Africa accounted for 42% and Southern Asia accounted for 37% of all such deaths [1].

The major causes that contribute to neonatal mortality in developing countries were prematurity, low birth weight, neonatal infections and birth asphyxia [3–5]. The first 48 hours following birth is very crucial period for newborn survival. In 2019 about 36% of neonates died within 24 hours, and up to three-quarters died in the first week of life [1, 6–8]. Even though the neonatal death rate reduced globally, this small reduction causes a significant barrier to achieve the Millennium Developmental Goal 4 [1].

The frequencies of the most common causes of neonatal deaths vary between regions [9–11]. Studies have shown that neonatal deaths are responsible for the majority of mortalities in children under the age of 5 years with a reported rate of 35% in the USA and up to 50% in some low-income countries [12]. The 2030 agenda for sustainable development by WHO calls for reduction in neonatal mortality rate to less than 12 per 1000 live births [7, 13]. There is a paucity of information regarding determinants of mortality for newborns in settings with the highest burden of neonatal deaths [1, 14].

Recently, global efforts to reduce neonatal mortality have focused primarily on community-based interventions largely because majority of births and deaths occur at home and access to facilities with capacity to manage newborns has been limited [8, 15–17]. These interventions have included training birth attendants to provide neonatal resuscitation, immediate care after delivery and encouraging perinatal hand washing [8, 18, 19].

In Eritrea neonatal mortality was 18 per 1000 live birth in 2019, reduced by almost 53% from 1990(34 death per 1000 live birth ) [1]. Study done in Neonatal Intensive Care Unit (NICU) at Orotta National Referral Hospital in Eritrea showed that the most common primary admission diagnosis were pneumonia (29%), sepsis (17%), dehydration (16%) and primary hypothermia (10%), and the overall neonatal mortality was 8.2% [20].

Neonatal death is one of the public health concerns worldwide including Eritrea as it is responsible for bulk of mortalities in children under the age of 5 years. So, determining the clinical profiles, outcomes and causes of admission will help the hospital to adjust the working protocols. Results of this research will also initiate the policy makers in extending the service to other hospitals and developing different guidelines regarding these issues.

## Methods and Materials

### Study area

Hospital Dekemhare is found in Dekemhare city, located 40 km South East of Asmara, the capital city of Eritrea. This hospital provides service to Dekemhare subzone, which has a population of around 60,000. It also serves as referral for three other subzones of Southern Region. In the hospital, there are different departments as medical, pediatric, maternity, dental, outpatient and ophthalmic. It also provides emergency cesarean section and maternal waiting room in addition to the NICU.

This NICU admits in and out born neonates with gestational age of greater than 28 weeks. It was established in 2017 and was staffed with one assigned physician and nine health assistants. During the study period, the NICU had capacity for thermoregulation, intravenous hydration, nasogastric tube feedings, limited phototherapy equipment, few number of oxygen concentrators for use with nasal prongs and electric supply with an automatic solar back up. Neonates requiring pediatric subspecialty care and surgery were transferred to the National Referral NICU center in Asmara.

### Study design and sample size

The study design was a retrospective register-based review of neonates admitted to Dekemhare Hospital NICU during the study period.. A total of 509 neonates were admitted to this NICU in these 4 years interval and all of them were enrolled in the study.

### Study period and population

The study was conducted in NICU of Dekemhare hospital from January 5 to February 5, 2022. All neonates who were admitted at NICU of Dekemhare hospital from January 2018 to December 2021 were the study population of this study. All neonates in the register book with full information were included in the study.

### Measurement variables

The dependent variable was neonatal outcome. The independent variables were gestational age, sex, residency, clinical diagnosis, length of hospital stay, mode of delivery and place of birth.

### Operational definitions

The neonatal period was considered from the day of birth up to 28 days of life. Prematurity was defined as a baby born before 37completed weeks of gestation and term refers a baby born after 37 complete weeks of gestation at the time of delivery. Neonatal intensive care unit was defined as a specialized section of a hospital that provides comprehensive and continuous care for neonates who are critically ill.

Neonatal sepsis was used as a type of neonatal infection and refers to the clinical presentation of infection in the setting of fever and/or hypothermia in a newborn baby. Death was defined as the cessation of all biological function that sustains an organism. Normal birth weight and low birth weight was used the following references, 2.5-4 kg and < 2.5 kg respectively. Neonatal mortality was defined as the number of neonatal deaths per1000 live births. Birth asphyxia is defined as an inability of newborn to initiate and sustain adequate respirations after delivery within 1st and 5th minutes of birth and ending with an Apgar score < 7 (2019 Woday et al.).

### Data collection and quality control

Data were collected from neonatal hospital register book from January 5 to February 5, 2022. This was done using a predesigned data extraction tool, which was used in other similar study. Data were collected by medical doctors of the hospital and training was given to them before the data collection time. Data quality was assured by pretesting in 5% of the sample size prior to the actual data collection in order to check the consistency and validity of data extraction tool on those who were admitted before the study period in the same hospital. Finally, the data collection tool was modified to the context and objectives of the study. During the actual data collection the investigators cross-checked 10% of the collected data from register book to revalidate the data collection tool and completeness of the data.

### Data entry and analysis

The obtained data were edited and entered in data entry screen developed using CSpro 7.3. Data analysis was carried out using SPSS version 22. Results were presented in frequencies, percent and odds ratio (crude and adjusted) with 95% confidence interval. Univariable and multivariable logistic regression were used to identify characteristics associated with mortality. Variables found to have statistically significant association with the outcome of interest at univariable analysis were considered in the multivariable logistic regression. All statistical tests were performed using two-sided tests at the 0.05 level of significance.

### Ethical considerations

Ethical clearance was obtained from the Research Ethics and Review Committee of Ministry of Health, and further permission was asked from offices of the Ministry of Health Debub branch and Dekemhare Hospital. All methods were performed in accordance with the relevant guidelines and regulations. Patient’s confidentiality was kept secured and only selected researchers accessed the data and personal identifiers were codded and removed from the analysis.

## Results

### Socio demographic characteristics of the neonates

A total of 509 neonates were enrolled with 54.8% males and 65.8% aged < 1 day at the time of admission to the NICU. Three fourth (75.6%) of the neonates had birth weight >  2.5 kg and 79% had gestational age > 37 weeks at delivery. Ninety-three percent were delivered at the health facilities and 65.2% were admitted to the NICU from the hospital delivery ward. Most (39%) of the neonates had stayed 4-7 days in the hospital and the leading causes of admissions were neonatal infection (33%), birth asphyxia (20%) and prematurity (14.3%). (Table 1).

**Table 1:** Baseline characteristics of the neonates with their outcome

Variable	Category	Neonatal outcome *N* = 509			
		Discharged n (%)^a^	Referred n (%)^a^	Death n (%)^a^	Total n (%)^b^
Residence	Rural	176 (76.2)	11 (4.8)	44 (19.0)	231 (45.4)
	Urban	227 (81.7)	12 (4.3)	39 (14.0)	278 (54.6)
Age on Admission (days)	< 1	260 (77.6)	12 (3.6)	62 (18.8)	335 (65.8)
	2-7	84 (84.8)	2 (2.0)	13 (13.2)	99 (19.4)
	8+	59 (78.7)	9 (12.0)	7 (9.3)	75 (14.7)
Gender	Female	186 (80.9)	10 (4.3)	34 (14.7)	230 (45.2)
	Male	217 (77.8)	13 (4.7)	49 (17.6)	279 (54.8)
Birth weight	> 2.5 kg	326 (84.7)	18 (4.7)	41 (10.6)	385 (75.6)
	< 2.5 kg	77 (62.1)	5 (4.0)	42 (33.9)	124 (24.4)
Gestational age at delivery	Preterm	68 (63.6)	4 (3.7)	35 (32.7)	107 (21.0)
	Term	335 (83.3)	19 (4.7)	48 (11.9)	402 (79.0)
Mode of delivery	Vaginal delivery	316 (76.7)	21 (5.1)	75 (18.2)	412 (80.9)
	CS delivery	87 (89.7)	2 (2.1)	8 (8.2)	97 (19.1)
Place of delivery	Home	20 (54.1)	5 (13.5)	12 (32.4)	37 (7.3)
	Health facility	383 (81.1)	18 (3.8)	71 (15.0)	472 (92.7)
Source of admission	Hospital delivery	270 (81.3)	11 (3.3)	51 (15.4)	332 (65.2)
	Referred from other facility	50 (67.6)	7 (9.5)	17 (23.0)	74 (14.5)
	From home	83 (80.6)	5 (4.9)	15 (14.6)	103 (20.2)
Duration of hospital Stay	< 1 day	10 (38.5)	3 (11.5)	13 (50.0)	26 (5.1)
	1-3 days	125 (69.8)	7 (3.9)	47 (26.3)	179 (35.2)
	4-7 days	178 (89.0)	7 (3.5)	15 (7.5)	200 (39.3)
	8 days +	90 (86.5)	6 (5.8)	8 (7.7)	104 (20.5)
Admission Diagnosis	Prematurity	47 (64.4)	1 (1.4)	25 (34.3)	73 (14.3)
	Birth asphyxia	68 (66.7)	5 (4.9)	29 (28.4)	102 (20.0)
	Neonatal infection	151 (89.9)	6 (3.6)	11 (6.5)	168 (33.0)
	Congenital abnormality	8 (38.1)	4 (19.0)	9 (42.9)	21 (4.1)
	Other conditions	130 (77.9)	7 (8.8)	9 (13.2)	145 (13.3)
Inborn / out born	Inborn	337 (82.8)	14 (3.4)	56 (13.8)	407 (80.0)
	Out born	66 (64.7)	9 (8.8)	27 (26.5)	102 (20.0)
Total		403 (79.2)	23 (4.5)	83 (16.3)	509 (100.0)

^a^ are row percentages; ^b^ are column percentages; *PROM* Premature Rupture Of Membrane

### Outcomes of neonatal admissions

**Fig. 1:**
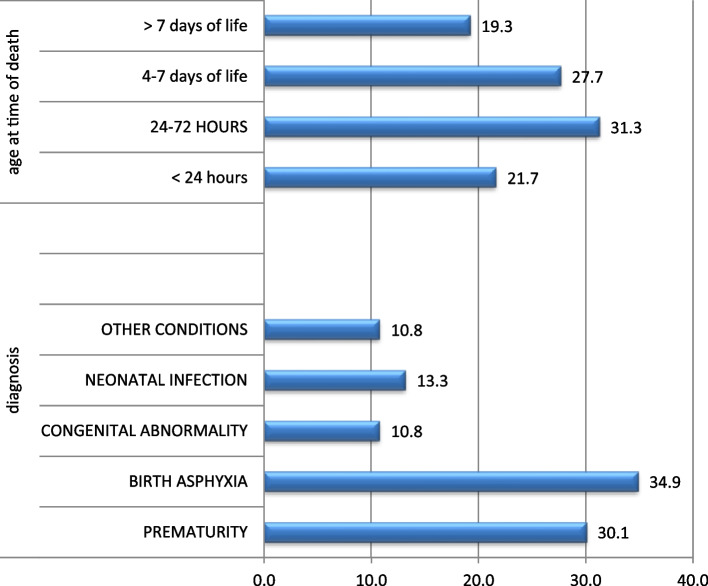
diagnosis and age at time of death neoanate in NICU dekemehare hospital, n (83)

Seventy-nine percent were discharged alive; besides, 4.5% were referred to a tertiary center in Asmara (Table 1). Overall neonatal hospital mortality rate was 16.3%. Of those who died, birth asphyxia was the commonest cause of admission (34.9%), followed by prematurity (30.1%) and neonatal infection (13.3%). (Fig. 1). But case fatality rate was high for neonates with congenital abnormality (43%) prematurity (34%) and birth asphyxia (28%). (Table 2).

**Table 2:** Predictors of neonatal hospital mortality at a univariable logistic regression analysis

Variables	Categories	Neonatal outcome *N* = 485^a^		COR (95%CI)	*P* value
		Discharged N (%)	Death N (%)		
Residence	Rural	178 (80.4)	44 (19.6)	1	
	Urban	227 (85.3)	39 (14.7)	0.7 (0.44-1.13)	0.09
Age on Admission (days)	< 1	260 (80.7)	62 (19.3)	1	
	2-7	84 (86.6)	13 (13.4)	0.65 (0.34-1.24)	0.190
	8-28	59 (89.4)	7 (10.6)	0.64 (0.24-1.69)	0.100
Gender	Female	186 (84.9)	34 (15.1)	1	
	Male	217 (81.6)	49 (18.4)	1.27 (0.79-2.06)	0.328
Birth weight	> 2.5 kg	328 (88.8)	41 (11.2)	1	
	< 2.5 kg	77 (65.3)	42 (34.7)	4.23 (2.57-6.97)	0.001
Gestational age at delivery	Preterm	68 (66.7)	35 (33.3)	1	
	Term	335 (87.5)	48 (12.5)	0.29 (0.17-0.48)	0.001
Mode of delivery	Vaginal delivery	316 (81.0)	75 (19.0)	1	
	CS delivery	87 (91.6)	8 (8.4)	0.39 (0.18-0.85)	0.017
Place of delivery	Home	20 (62.5)	12 (37.5)	1	
	Health facility	383 (84.5)	71 (15.5)	0.31 (0.14-0.65)	0.002
Source of admission	Hospital delivery	270 (84.1)	51 (15.9)	1	
	Referred from other facility	50 (75.8)	17 (24.2)	1.69 (0.90-3.21)	0.105
	From home	83 (84.7)	15 (15.3)	0.96 (0.51-1.79)	0.890
Duration of hospital Stay	< 1 day	10 (43.5)	13 (56.5)	1	
	1-3 days	125 (72.7)	47 (27.3)	0.29 (0.12-0.7)	0.006
	4-7 days	178 (92.2)	15 (7.8)	0.07 (0.02-0.17)	< 0.001
	8 days +	90 (92.8)	8 (7.2)	0.06 (0.02-0.19)	< 0.001
Diagnosis	Prematurity	47 (66.2)	25 (33.8)	1	
	Birth asphyxia	68 (70.1)	29 (29.9)	0.84 (0.43-1.61)	0.591
	Neonatal infection	164 (93.7)	11 (6.3)	0.14 (0.07-0.31)	< 0.001
	Congenital abnormality	8 (47.1)	9 (52.9)	2.2 (0.75-6.44)	0.149
	Other conditions	189 (93.3)	8 (6.7)	0.33 (0.14-0.79)	0.012
Inborn/ out born	Inborn	337 (85.8)	56 (14.2)	1	
	Out born	66 (71.7)	27 (28.3)	2.37 (1.39-4.05)	0.001
Total		403 (83.1)	82 (16.9)		

a 23 cases were not included in the analysis (referred out cases)

### Association of neonate’s background characteristics to their outcomes

Fifty percent of deaths occurred on those who stay < 24 hours in the hospital. (Table 1) Being term (OR: 0.29, 95%CI 0.17-0.48), cesarean section delivery (OR: 0.39, 95%CI 0.18-0.85) and delivery at health facilities (OR: 0.31, 95%CI 0.14-0.65) had a protective effect in the neonatal outcome. (Table 3).

**Table 3:** Predictors of neonatal hospital mortality at a multivariable logistic regression analysis

Variable	Categories	AOR (95% C.I.)	*P* value
Place of delivery	Home or on the way to health facility	1	
	Health facility	0.17 (0.06-0.471)	< 0.001
Duration of hospital stay	0	1	
	1-3 days	0.32 (0.10-1.03)	0.057
	4-7 days	0.06 (0.02-0.23)	< 0.001
	8 and above	0.01 (0.00-0.06)	< 0.001
Diagnosis	Prematurity	1	
	Birth asphyxia	0.45 (1.53-0.51)	0.454
	Neonatal infection	0.10 (0.36-0.11)	0.101
	Congenital abnormality	0.14 (3.25-0.68)	0.139
	Other conditions	1.12 (0.00-0.00)	1.0
Birth weight (kg)	> 2.5	1	
	< 2.5	7.28 (2.85-18.55)	< 0.001

In univariable analysis, low birth weight, preterm delivery, neonatal infection, home delivery, hospital stay < 1 day, vaginal delivery and out born were found to have statistically significant association with neonatal mortality. (Table 2) In multivariable analysis home delivery, hospital stay < a day, and low birth weight were retained as the lone predictor of neonatal mortality (*p* value < 0.05).(Table 3).

The odds of having neonatal death was reduced by 80% (AOR: 0.17; 95%CI: 0.06–0.471) among neonate who delivered at health facilities compared to those delivered at home. Similarly, neonates who stayed in hospital for 4-7 days and above 8 days had 94% (AOR: 0.06; 95%CI: 0.02–0.23) and 99% (AOR: 0.01; 95%CI: 0.00-0.06) reduced morality risk than those hospital stay < a day respectively. The odds of neonatal death was 7 times greater (AOR: 7.28; 95%CI: 2.85-18.55) among low birth weight than their counterparts (Table 3).

## Discussion

Majority of the neonates had NBW (75.6%) and were term (79.0%) at delivery. This was higher to study done in Cameron, NBW (53.9%) and term (66.5%) [21], central India, NBW (38.4%) and term (49% )[22], Jordan NBW (52%) and term (42.4%) [23], Uganda NBW (53.9%) and term (60%) [6], Iran NBW (23.5%) and term (4.4%) [24] and South Africa NBW (47.5%) and term (56.3%) [25]. This higher rate of NBW and term at delivery in this study decreases the neonatal mortality of the country compared to the other countries. This is mainly due to preterm and LBW infants are predisposed to different complications. But this was lower to a study done in the National NICU and the neonatal mortality was proportionally higher in this study.

Our study indicated that 81% of the neonates were delivered vaginally and 19% were delivered by cesarean section. The cesarean section rate was lower than studies done in Uganda, 46% [6], Jordan, 64 % [23] and South Africa,33.3 % [25]. But our result was higher than studies done in Somali region of Ethiopia, 7.3% [26] and Central India, 11.6 % [27]. And, the cesarean section rate was similar in study done in Eritrea, 22.2 % [20]. This result of cesarean section rate coincides with the previous study in Eritrea and higher than the WHO recommendation of cesarean section (5 to 15%). This higher rate of cesarean section in other studies could be mainly due to that they may include other indications as cesarean section on maternal request which could increase the cesarean section rate.

This study revealed that 7.3% of neonates were delivered at home and they had mortality rate of 14.5% (12/83). This was higher than study done in Central India, 2.21% were delivered at home and a mortality of 2.7% [27] and Ethiopia,3.3% delivered at home and 2.3% mortality rate [28]. Sensitization and increasing awareness on the community and interventions and usage of maternity waiting home were vital to decrement of neonates delivered at home as they have higher morbidity and mortality [29]. Even though the Government of Eritrea had made remarkable contribution to reduce home delivery by community sensitization and building of waiting homes in different hospitals of the country, still community awareness is crucial as neonates delivered at home have higher morbidity and mortality.

Based on this study, 39.3% of the neonates have stayed 4-7 days in the hospital and the highest rate of neonatal death had occurred during the first 3 days of their admission (73%, 60/83). Similarly, other study showed that the highest rate of neonatal death occurred during the first 3 days of their admission (38.3-49.7% )[25, 28]. Similarly, study done by Engmann et al. showed that over 80% of early neonatal death occurred during the first 3 days of postnatal life [4]. In our study, early neonatal deaths (< 7 day’s age) were 62.3% (75/83) which was lower than a study done in Ethiopia (97.7% )[28]. This higher rate of early neonatal age death depicts that the higher vulnerability of the neonates as the number of prematurity, infection and birth asphyxia was high [4, 14]. This also showed that the effects of the neonatal interventions done at that neonatal age are lifesaving and should be encouraged, like increase birth skilled attendance and birth at health facility [14]. As neonates with prematurity, congenital abnormality and these with severe asphyxia were the most common cause of death, this is expected to happen in their early neonatal age and death was inevitable in most cases as to the capacity of the hospitals NICU.

This study indicated that, neonatal infection, birth asphyxia and prematurity, was the top causes of admission of neonates to the NICU. In the same way, other studies showed that, infection, prematurity, respiratory distress and asphyxia were among the main factors for neonatal admission [6, 10, 22, 23, 25, 27, 30–32]. But, it was different from a study done in the NICU of National Referral Hospital of Eritrea; where the most common primary admission diagnosis was pneumonia, sepsis, dehydration and hypothermia [20].

This study specified that the mortality rate of neonates admitted to the NICU was 16.3%. This was higher to other studies whose overall mortality rate was 3.8, 8.93, 8.7, 11, 7.5, 12.5% [22–24, 28, 31, 32]. Furthermore, this result was higher to studies conducted in the NICU at Orotta National Referral Hospital in Eritrea,where the neonatal mortality was 8.2% and that of the overall neonatal mortality in Eritrea was 18 per 1000 live birth in 2019 [1, 20]. But it was similar to study done in Cameron (15.7%) and lower than study done in Central India (31.8%) [21, 25, 27]. The higher mortality rate in the NICU could be mainly due to the difference in diagnostic setup and treatment tools, infrastructure setting, neonatal resuscitation experiences and human power setup compared to the national level and other countries. As this NICU is newly established, it had no incubators, chemistry machine, blood exchange transfusion, emergency pediatric surgery and had no pediatrician as to the national NICU. Thus, higher mortality could be expected for preterm and severely asphyxiated neonates who need complex urgent management.

This study reports that birth asphyxia, prematurity and neonatal infection was the most common causes of neonatal mortality which was similar to other studies done in Uganda, South Africa, Cameron, central India, North India, Jordan, Ghana, Namibia, Somalia region of Ethiopia [6, 14, 21, 22, 25–27, 30–34]. Most of these causes are inevitable and can be prevented in well-equipped infrastructure, manpower, intrapartum and neonatal resuscitation practices that can prevent birth asphyxia.

This study added that even though the government of Eritrea made remarkable efforts to decrease the neonatal death by establishment of NICU in different hospitals of the country and introducing emergency maternal care and neonatal resuscitation to the level of hospitals, still the neonatal deaths due to inevitable causes as prematurity and congenital anomalies is higher in this hospital. This needs prospective study to find further determinants of neonatal death. The causes of neonatal death and prevalence of cesarean section delivery was almost similar to previous national studies and WHO estimates of cesarean section.

### Limitation of the study

The main limitation of this study, was that being a retrospective some important data were missed and cannot be analyzed, and also it was a single center study, so the finding cannot be generalized outside the study area.

## Conclusion

Congenital abnormality**,** prematurity and birth asphyxia had higher case fatality rate in this study. And, home delivery, hospital stay < a day, and low birth weight were retained as the lone predictor of neonatal hospital mortality. Neonatal mortality was higher and cesarean section rate was almost similar to previous studies done in the country.

### Recommendations

Raising community awareness at health facility delivery and establishment of waiting rooms for mothers is important first step to decrease the neonatal mortality. Regular antenatal care should be encouraged to all pregnant mothers in raising awareness on complications of delivery and neonatal outcomes. Equipping the available NICU in terms of manpower and equipment’s are also crucial. Extending the NICU service to hospital where there is no pediatrician and raising awareness of health professionals on neonatal resuscitation are key areas in the improvement of the neonatal outcome. Further prospective studies with larger sample size and geographical settings are highly recommended.

## Data Availability

The datasets generated and/or analyzed during the current study are available from the corresponding author on reasonable request.

## Abbreviations

NICU: Neonatal intensive care unit
CSPro: Census and Survey Processing System
SPSS: Statistical Package for the Social Sciences
PROM: Premature Rupture of Membrane
NBW: Normal Birth Weight
